# A diagnostic challenge; pelvic solitary fibrous tumor (SFT) mimicking Peri‐Anal gastrointestinal stromal tumor (GIST): A case report

**DOI:** 10.1002/ccr3.7666

**Published:** 2023-07-10

**Authors:** Seyed Amir Miratashi Yazdi, Payvand Parhizkar Roudsari, Hadi Ahmadi Amoli, Reza Hajebi

**Affiliations:** ^1^ Department of Surgery Sina Hospital, Tehran University of Medical Sciences Tehran Iran

**Keywords:** gastrointestinal stromal tumor, immunohistochemistry, pelvic mass, solitary fibrous tumor

## Abstract

**Key Clinical Message:**

The study reports a rare case of pelvic solitary fibrous tumor (SFT) that was initially considered as a peri‐anal gastrointestinal stromal tumor due to similar radiologic/pathologic features. SFT diagnosis can be challenging due to its rarity and wide range of diagnoses that must be ruled out precisely.

**Abstract:**

Solitary fibrous tumors (SFTs) are rare tumors that can occur in any part of the body. Although usually benign, malignant SFTs have been reported, especially outside the lungs. Radiology can help with diagnosis, but immunohistochemistry is necessary to distinguish SFTs from other possible diagnoses such as gastrointestinal stromal tumors (GISTs). This study presents a rare case of pelvic SFT initially considered to be a peri‐anal GIST, highlighting the importance of accurate diagnosis given the rarity of SFTs and the need to rule out other potential diagnoses.

## Graphical abstract

Resected pelvic solitary fibrous tumor (SFT).

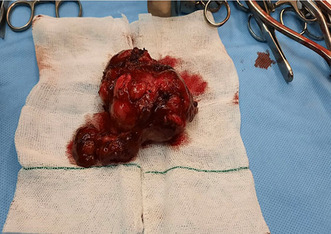

## 1 INTRODUCTION

Solitary fibrous tumor (SFTs) are mesenchymal neoplasms with a low incident rate of about 0.35 in 100,000 persons.^1^ They can develop in any organ but barely have gastric origins.^2^, ^3^ The anal margin, pelvis, and ischiorectal fossa are also exceedingly rare regions for SFT occurrence.^4^, ^5^, ^6^ On the other side, gastrointestinal stromal tumor (GISTs) can be mentioned as other rare neoplasms accounting for about 1% of gastrointestinal (GI) tumors.^7^ They are the most prevalent mesenchymal neoplasms that can be found in all over the GI tract, especially in the stomach, esophagus, and anorectum sites. The Anorectum region is responsible for 5% of GIST cases in which the anal canal contains about 2%–8% of them.^8^, ^9^ Mesentery and omentum could be also noted as the unusual areas for GIST development.^10^

Most GISTs have spindle‐cell type configuration that SFTs should be considered as a notable differential diagnosis for spindle‐cell type GISTs.^11^ Although radiologic assessments (like computed tomography (CT) scans) are beneficial tools for detecting both GISTs and SFTs, they have some diagnostic limitations because GISTs and SFTs overlap in some of their characteristics like anatomic location.^4^, ^12^ Immunohistochemistry (IHC) staining can significantly aid accurate diagnosis in order to differentiate GIST from SFT. For instance, CD117‐c‐kit proto‐oncogene protein is mentioned as the specific and sensitive indicator of GISTs which represents a strong distinguishing feature between GIST and SFT as it is negative in almost all of the SFTs.^11^, ^12^, ^13^ On the other hand, nuclear STAT6 is a strongly positive marker for SFTs.^11^ It should be also mentioned that GIST and SFT may also overlap in some IHC‐related markers like CD34, emphasizing the necessity of a more accurate diagnosis.^11^, ^12^, ^14^ Herein, due to the importance of the exact diagnosis of GIST and SFT regarding the remaining diagnostic challenges^12^ in addition to the extremely rare incidence of both pelvic SFT and peri‐anal GIST, we have provided a case report of a hypervascular pelvic SFT that first was suspected as a peri‐anal GIST.

## 2 CASE REPORT

A 59‐year‐old woman (having a past medical history of diabetes mellitus and hypertension) presented to the hospital with radiology reports containing pelvic mass. The patient had no significant symptoms except slight generalized abdominal pain and peri‐anal discomfort and she did not state any previous experience of similar abdominal or pelvic pain and GI disorder in herself or her family members. All of the examinations including abdominal and rectal examinations were normal without any suspicious findings.

All of the patient's medical data regarding CT scan, magnetic resonance imaging (MRI), sonographies, and angiography prior to angioembolization in addition to the final pathology and IHC results of the resected tumor, are provided in order in the following parts.

**FIGURE 1:**
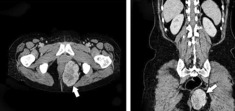
Spiral Thoraco‐abdomino‐pelvic CT Scan. A 96 × 66 × 48 mm mass on the left peri‐anal site growing to the perineal region. Arrows indicate the mass on the left perianal region.

**FIGURE 2:**
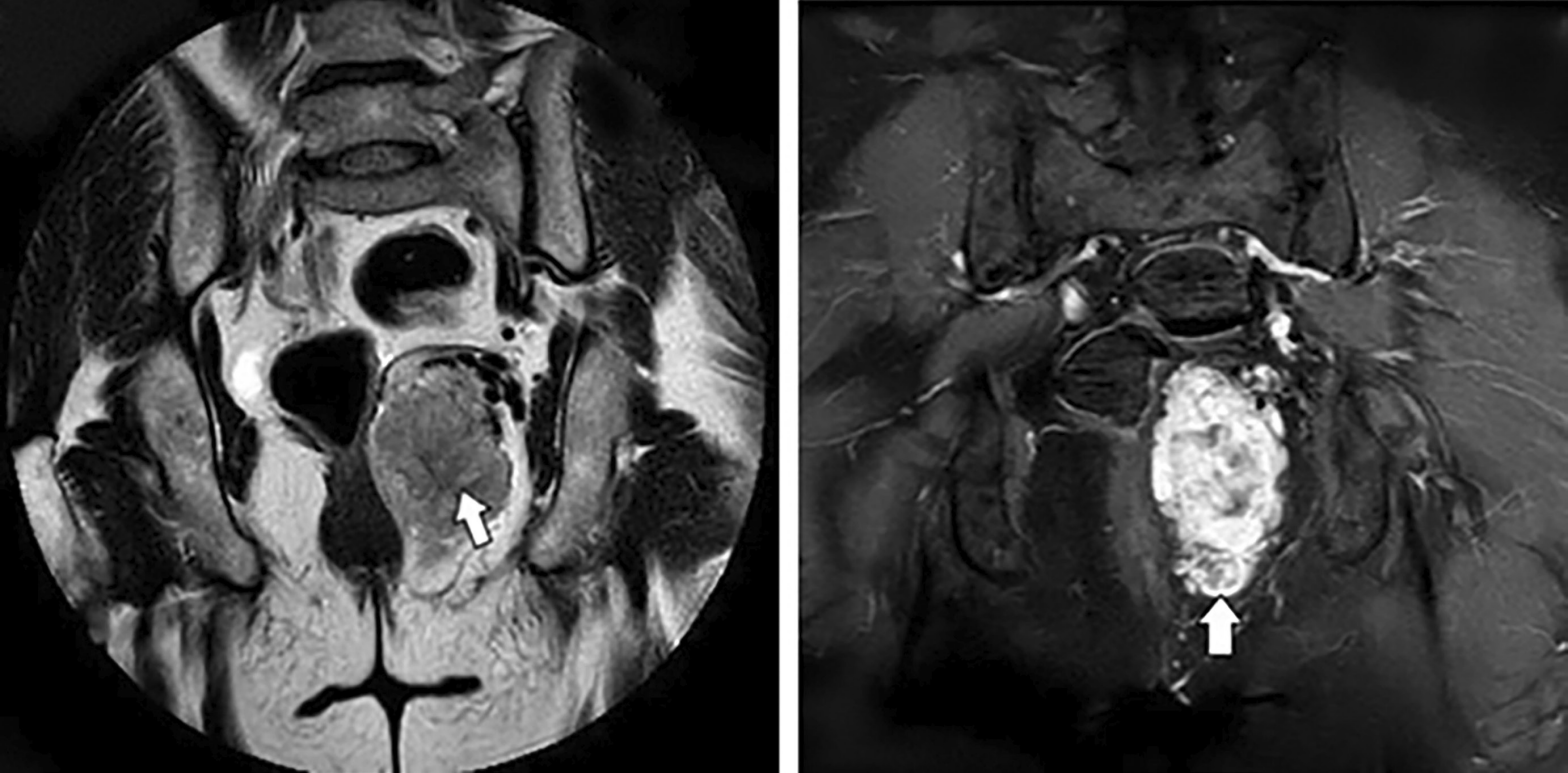
Pelvic MRI. Arrows indicate a large enhancing T2 signal intensity lesion in the left side of peri‐anal soft tissue.

**FIGURE 3:**
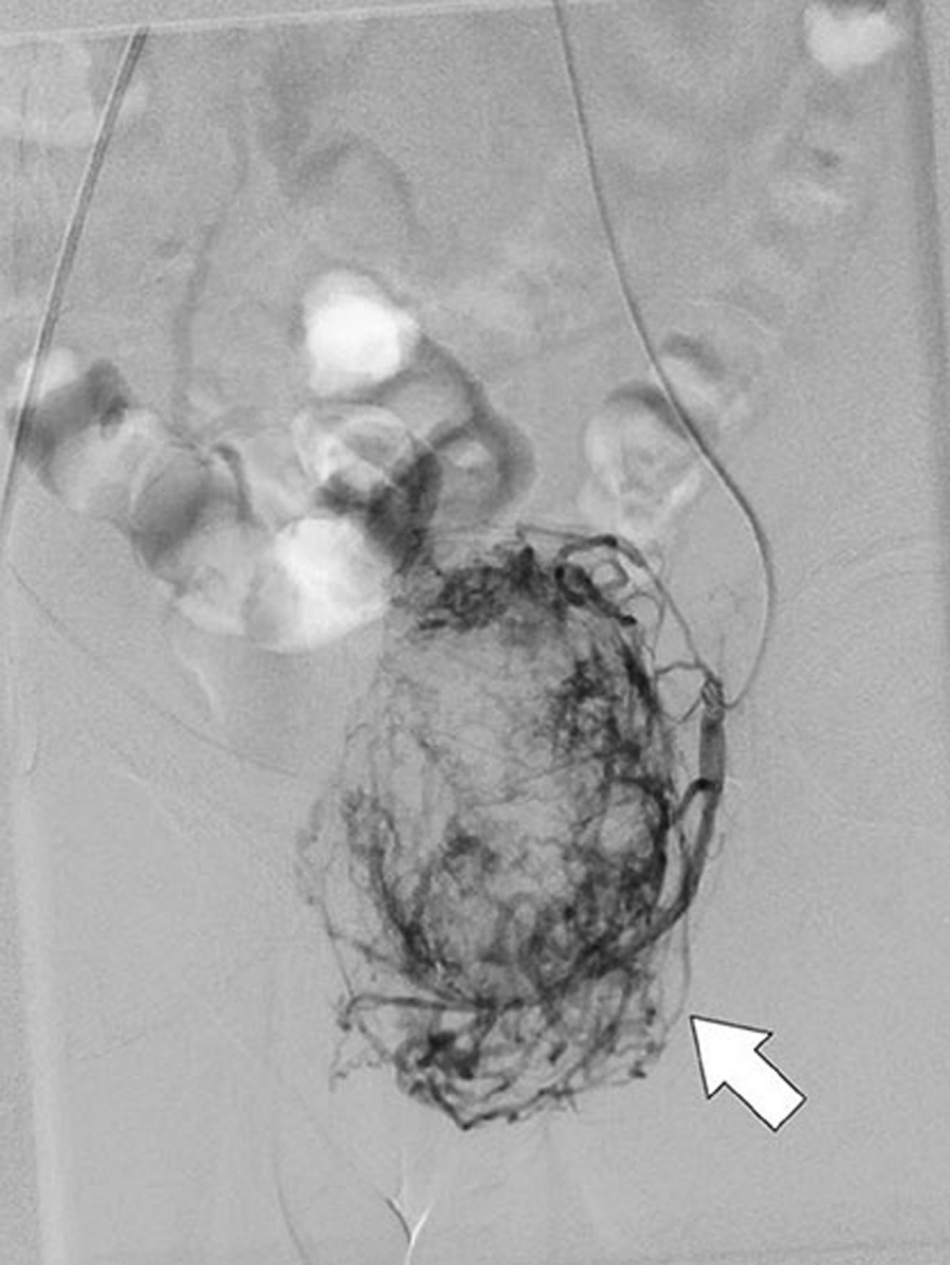
Left limb angiography. A hypervascular mass (arrow).

First, a spiral thoraco‐abdomino‐pelvic CT scan with and without contrast was performed in which an infiltrative mass with the central necrotic components was found in the left peri‐anal region pressing the external anal sphincter. The mass had the size of about 96 × 66 × 48 mm growing to the perineal area causing superior displacement of levator ani on the left side; still, it had not shown any developments to the peri‐rectal region and is limited to the perineal region. Blood supply of the mass was provided by the left pudendal artery. No invasion to the muscles, bones, and other peripheral structures was mentioned and all other organs and structures were completely intact (Figure 1). After that, the patient underwent pelvic MRI to find out the tumors particular characteristics. Pelvic MRI results revealed a large and severely enhancing T2 signal intensity mass lesion in the left side of peri‐anal soft tissue with some similar characteristics to those on CT scan in addition to tortoise dilation of the left pudendal artery. Soft tissue sarcoma or malignant nerve sheath tumor are suggested as probable diagnoses (Figure 2). Peri‐anal sonography was also done in order to confirm the results. The hypoechogenic ischiorectal mass was noted with non‐specific sonographic findings tended to be a neoplastic mass. Sono‐guided needle biopsy was also carried out that reported a spindle‐cell tumor containing eosinophilic cytoplasm and rare mitosis without nuclear atypia and necrosis. Findings were compatible with GIST (low‐risk type) due to the results. Digital angiography of the aorta and iliac arteries and left lower limb revealed a hypervascular mass with left internal iliac origin. According to the hypervascularity of the noted mass, angioembolization was considered a prior approach to complete resection (Figure 3).

**FIGURE 4:**
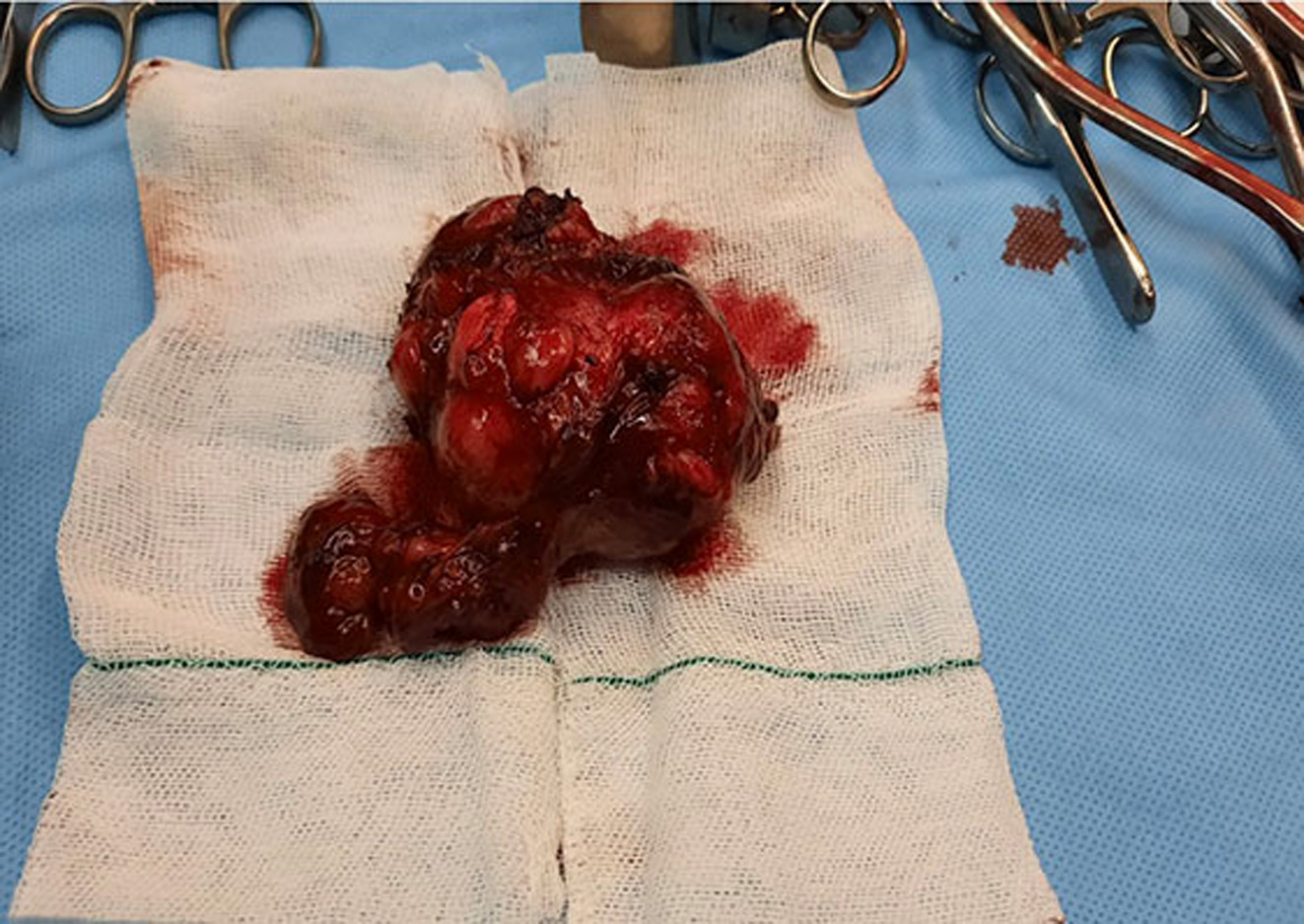
Resected pelvic solitary fibrous mass (SFT).

After complete tumor resection (Figure 4), the pelvic mass underwent pathologic analyses, including IHC assessments. STAT6 and S100 were strongly positive in tumor cells; whereas, CD117, pancytoleratin and desmin were negative markers. CD34, BCL2, and TLE1 were also positive in tumor cells. Altogether, according to the histomorphologic features and IHC study, SFT (on Grade II) was suggested as a probable diagnosis.

## 3 DISCUSSION

SFT is a spindle‐cell fibroblastic mesenchymal neoplasm that can affect almost all anatomic sites but most commonly occurs in visceral pleura.^15^ Although, 50%–70% of SFTs are mentioned to originate from outside the thorax.^16^ Herein, pelvic SFT is an extremely rare tumor, usually larger along with more malignant behaviors.^3^ In the current study, we have also reported a rare case of grade II pelvic SFT. SFTs typically affect men and women equally and in middle ages^5^ with no specific symptoms typically until the tumor starts to compress peripheral structures.^3^ It is similar to our study in that the patient has no significant complaints except some discomforts in her abdomen and peri‐anal region. Although, the CT scan showed the compressing signs of the tumor on the external anal sphincter. Many of the SFTs are discovered on imaging incidentally due to their asymptomatic and non‐specific features.^17^ The neoplasm has a well‐circumscribed or encapsulated morphology consisting of ovoid/spindle‐cells in a patternless distribution without clear atypia along with rare mitotic features. However, malignant SFT is associated with more mitotic pleomorphic characteristics in addition to hemorrhage and necrosis findings.

In addition to the rarity of this neoplasm, SFT has a wide range of differential diagnoses that make accurate SFT diagnosis more challenging. Indeed, imaging studies along with considering related clinical features may lead to SFT diagnosis; but a definite diagnosis should be confirmed through IHC evaluations in order to differentiate it from other mesenchymal tumors with similar sources.^15^, ^16^ CD34 and Bcl‐2 are the most considerable positive SFT indicators in the first steps. Challenging cases can also benefit from STAT‐6 detection with high sensitivity and specificity for diagnosing SFTs.^18^ Herein, we discovered the large severely enhancing T2 signal intensity mass that first was suspected as a peri‐anal GIST according to initial reports. But it turns out to be a SFT regarding the IHC report after complete resection. GI SFTs should be precisely distinguished from four major types of GI tract neoplasms including GIST besides desmoid type fibromatosis, leiomyosarcoma, and schwannoma. GISTs are rare tumors with mesenchymal origins that can be seen anywhere in the GI tract leading to some misdiagnoses, particularly in pelvic mass presenting.^19^ In addition to mentioned IHC markers related to SFT, CD117 is the most powerful GIST's distinguishing indicator since it is a negative marker in almost all of the SFTs.^18^ Also, Discovered On GIST‐1 (DOG1) has been introduced as an encouraging diagnostic marker of GISTs with high sensitivity/specificity irrespective of CD117 expression.^20^ Some other studies have also reported the rare SFT masses in which accurate diagnosis benefited from IHC findings significantly. For instance, in the study by Rekhi and colleagues, the diagnosis of a rare vaginal SFT (with a complex cystic appearance) was confirmed through STAT6 immunostaining. They also insist on the importance of distinguishing SFT from its mimics to perform the accurate treatment method.^21^ IHC markers also helped to differentiate SFT from its differential diagnoses in a study by Rao et al. in which a child was presented with a congenital mass.^22^ Guo et al. showed that histological tests in addition to gene sequencing could help to distinguish a malignant SFT of the greater omentum which was first suspected as a GIST.^23^ Lau and colleagues also showed the importance of IHC methods in SFT diagnosis by providing a rare SFT case (with small intestinal mesentery origin) which has some similarities to a GIST on imaging.^24^

Complete resection is the choice treatment strategy for SFTs that is connected to patients' prognosis^5^, ^6^ as our patient showed no recurrence or metastasis at least until the fourth year of follow‐up. However, long‐term and close follow‐up visits are essential due to the existing risk of malignant transformations or recurrence. It has been stated that larger (above 10 cm) or extrathoracic SFTs, positive margins, and malignant components place patients at a higher risk for recurrence.^5^ Some studies have also mentioned the effectiveness of the postoperative radiotherapy and chemotherapeutic agents for controlling SFT progression.^3^ Moreover, regarding the vascular nature of SFTs, preoperative interventional embolization of the tumors' feeding vessel should be considered which can result in decreased intraoperative bleeding and surgery duration.^6^ Our study has also benefited from preoperative angioembolization of the hypervascular mass, although it was first thought to be a GIST.

## 4 CONCLUSION

SFT can be introduced as a rare and slow‐growing metastasizing fibroblastic mesenchymal neoplasm having a variety of differential diagnoses and can mimic other tumors' characteristics. Although its diagnostic processes may benefit from radiologic evaluations in the first steps, IHC studies have shown great importance in distinguishing it from other diagnoses due to diagnostic challenges.^16^, ^17^, ^18^ It insists on the necessity of a more precise diagnosis which was explained through a rare hypervascular SFT case in this article that was thought to be a GIST in earlier steps.

## AUTHOR CONTRIBUTIONS

**Seyed Amir Miratashi Yazdi:** Conceptualization; writing – review and editing. **Roudsari Payvand Parhizkar:** Conceptualization; writing – original draft; writing – review and editing. **Hadi Ahmadi Amoli:** Conceptualization; supervision. **Reza Hajebi:** Conceptualization; writing – review and editing.

## FUNDING INFORMATION

This study received no funding or financial support.

## CONFLICT OF INTEREST STATEMENT

The authors declare that there is no conflict of interest.

## ETHICS STATEMENT

The study was performed regarding the ethics committee of Tehran University of Medical Sciences.

## CONSENT

The authors have confirmed that patient consent has been signed and collected in accordance with the journal's patient consent policy.

## Data Availability

All data and materials on which the paper's conclusions rely be presented in the main paper.
